# Supplementary material for: Bondarzewia dickinsii Against Colitis-Associated Cancer Through the Suppression of the PI3K/AKT/COX-2 Pathway and Inhibition of PGE2 Production in Mice
Source: Nutrients. 2024 Nov 26;16(23):4048. doi: 10.3390/nu16234048 (PMC11643906; doi:10.3390/nu16234048)
Supplement: Supplementary file 1 [file nutrients-16-04048-s001.zip › nutrients-3313708-supplementary.pdf]

Bondarzewia dickinsii against colitis-associated cancer by the suppression of the PI3K/AKT/COX-2 pathway and inhibition of PGE2 production in mice

Junliang Chen [1, 2], Shuai Liu [1], Xin Zhang [1], Xiaojing Dai [1], Yu Li [1, 3, 4], Yonglin Han [5], Lanzhou Li [1,*]

[1] Engineering Research Center of Edible and Medicinal Fungi, Ministry of Education, Jilin Agricultural University, Changchun, 130118, China; 20221377@mails.jlau.edu.cn, 2021200116@stu.syau.edu.cn, daixiaojing@mail.jlau.edu.cn, liyu@jlau.edu.cn

[2] Science and Research Center for Edible Fungi of Qingyuan County, Qingyuan, 323800, China; chenjunliang1026@126.com

[3] Tianjin Institute of Industrial Biotechnology, Chinese Academy of Sciences, Tianjin, 300308, China

[4] National Center of Technology Innovation for Synthetic Biology, Tianjin, 300308, China

[5] Science Popularization Service Center of Jilin Province, Changchun, 130021, China; 2030553021@qq.com

* Correspondence: lilanzhou@jlau.edu.cn;

Table S1. Analysis Methods on the content of regular nutritive components in *Bondarzewia dickinsii.*

Compounds	Methods
Total sugar	The phenol-sulfuric acid determination
Reducing sugar	3,5-dinitrosalicylic acid colorimetric estimation
Triterpenoids	Vanillin-glacial acetic acid and perchloric acid colorimetric spectrophotometry
Flavonoids	The aluminium chloride colorimetric method
Mannitol	The periodate oxidation method
Crude fat	The petroleum benzine extraction method
Total ash	The ashing method
Total protein	The Kjeldahl method
Crude fiber	Enzymatic hydrolysis method
Sterols	Liebermann-Burchard reagent
Polyphenols	Folin-Ciocalteu method

Table S2. Dominant nodes based on LEfSe analysis of intestinal microbiota in CAC mice.

Group	Taxa	Abundace (log_{10})	LDA score	*P*
BD	Bacteria.Proteobacteria.Gammaproteobacteria.Pseudomonadales.Moraxellaceae.*Acinetobacter*	2.002	2.992	0.022
	Bacteria.Bacteroidetes.Bacteroidia.Bacteroidales.Bacteroidaceae.*Bacteroides.Bacteroides_ovatus*	3.339	3.049	0.005
	Bacteria.Proteobacteria.Gammaproteobacteria.Pseudomonadales	2.297	3.147	0.007
	Bacteria.Firmicutes.Bacilli.Turicibacterales.Turicibacteraceae	3.186	2.897	0.015
	Bacteria.TM7.TM7_3	3.509	3.171	0.009
	Bacteria.TM7.TM7_3.CW040	3.509	3.171	0.009
	Bacteria.Tenericutes.Mollicutes.RF39	4.033	3.638	0.035
	Bacteria.TM7.TM7_3.CW040.F16	3.509	3.171	0.009
	Bacteria.TM7	3.509	3.171	0.009
	Bacteria.Firmicutes.Bacilli.Turicibacterales.Turicibacteraceae.*Turicibacter*	3.186	2.898	0.015
	Bacteria.Firmicutes.Bacilli.Turicibacterales	3.186	2.897	0.015
	Bacteria.Proteobacteria.Gammaproteobacteria.Pseudomonadales.Moraxellaceae	2.002	2.978	0.041
	Bacteria.Bacteroidetes.Bacteroidia.Bacteroidales.Prevotellaceae	4.293	3.939	0.031
	Bacteria.Bacteroidetes.Bacteroidia.Bacteroidales.Prevotellaceae.*Prevotella*	4.285	3.941	0.021
Ctrl	Bacteria.Firmicutes.Clostridia.Clostridiales.Dehalobacteriaceae	3.529	3.216	0.032

Bacteria.Firmicutes.Clostridia.Clostridiales.Ruminococcaceae	4.729	4.265	0.018
Bacteria.Firmicutes.Erysipelotrichi.Erysipelotrichales.Erysipelotrichaceae.*Clostridium.Clostridium_cocleatum*	2.665	2.633	0.032
Bacteria.Firmicutes.Clostridia.Clostridiales.Ruminococcaceae.*Butyricicoccus.Butyricicoccus_pullicaecorum*	2.081	3.04	0.007
Bacteria.Firmicutes.Clostridia.Clostridiales.Ruminococcaceae.*Butyricicoccus*	2.081	3.04	0.007
Bacteria.Proteobacteria.Gammaproteobacteria.Enterobacteriales	2.826	2.653	0.048
Bacteria.Firmicutes.Clostridia.Clostridiales.Ruminococcaceae.*Ruminococcus*	4.174	3.796	0.007
Bacteria.Bacteroidetes.Bacteroidia.Bacteroidales.Rikenellaceae.*AF12*	3.237	2.929	0.038
Bacteria.Firmicutes.Clostridia.Clostridiales.Ruminococcaceae.*Oscillospira*	4.428	4.084	0.005
Bacteria.Bacteroidetes.Bacteroidia.Bacteroidales.Rikenellaceae	4.381	3.977	0.007
Bacteria.Firmicutes.Erysipelotrichi.Erysipelotrichales.Erysipelotrichaceae.*Clostridium*	2.675	2.69	0.032
Bacteria.Bacteroidetes.Bacteroidia.Bacteroidales._Odoribacteraceae_.*Odoribacter*	3.512	3.22	0.005
Bacteria.Proteobacteria.Gammaproteobacteria.Vibrionales.Vibrionaceae.*Vibrio*	2.331	2.849	0.032
Bacteria.Proteobacteria.Gammaproteobacteria.Vibrionales	2.386	2.817	0.016
Bacteria.Tenericutes.Mollicutes.Anaeroplasmatales	2.965	2.671	0.018
Bacteria.Gemmatimonadetes	1.622	3.284	0.047
Bacteria.Bacteroidetes.Bacteroidia.Bacteroidales._Odoribacteraceae_	3.692	3.274	0.039
Bacteria.Bacteroidetes.Bacteroidia.Bacteroidales._Paraprevotellaceae_._Prevotella_	4.666	4.252	0.015

	Bacteria.Proteobacteria.Gammaproteobacteria	2.992	2.593	0.036
	Bacteria.Proteobacteria.Gammaproteobacteria.Enterobacteriales.Enterobacteriaceae	2.826	2.653	0.048
	Bacteria.Tenericutes.Mollicutes.Anaeroplasmatales.Anaeroplasmataceae	2.965	2.667	0.018
	Bacteria.Bacteroidetes.Bacteroidia.Bacteroidales._Paraprevotellaceae_	4.666	4.252	0.015
	Bacteria.Tenericutes.Mollicutes.Anaeroplasmatales.Anaeroplasmataceae.*Anaeroplasma*	2.965	2.669	0.018
	Bacteria.Firmicutes.Clostridia.Clostridiales.Dehalobacteriaceae.*Dehalobacterium*	3.529	3.216	0.032
	Bacteria.Proteobacteria.Gammaproteobacteria.Vibrionales.Vibrionaceae	2.331	2.849	0.032
Model	Bacteria.Actinobacteria.Actinobacteria.Bifidobacteriales.Bifidobacteriaceae.*Bifidobacterium.Bifidobacterium_pseudolongum*	3.265	2.97	0.006
	Bacteria.Proteobacteria.Gammaproteobacteria.Enterobacteriales.Enterobacteriaceae.*Serratia*	2.514	2.708	0.017
	Bacteria.Fusobacteria.Fusobacteriia.Fusobacteriales.Leptotrichiaceae	1.963	3.061	0.032
	Bacteria.Fusobacteria.Fusobacteriia.Fusobacteriales.Leptotrichiaceae.*Sneathia*	1.963	3.071	0.032
	Bacteria.Firmicutes.Clostridia.Clostridiales.Ruminococcaceae.Anaerotruncus	1.901	2.91	0.034

Data are presented as the mean, (n = 4) and analyzed via a one-way ANOVA test followed by post-hoc Dunn’s multiple comparison tests. The abundance of dominant nodes was provided with logarithm (log_{10}). LDA score and *P* value were also provided.

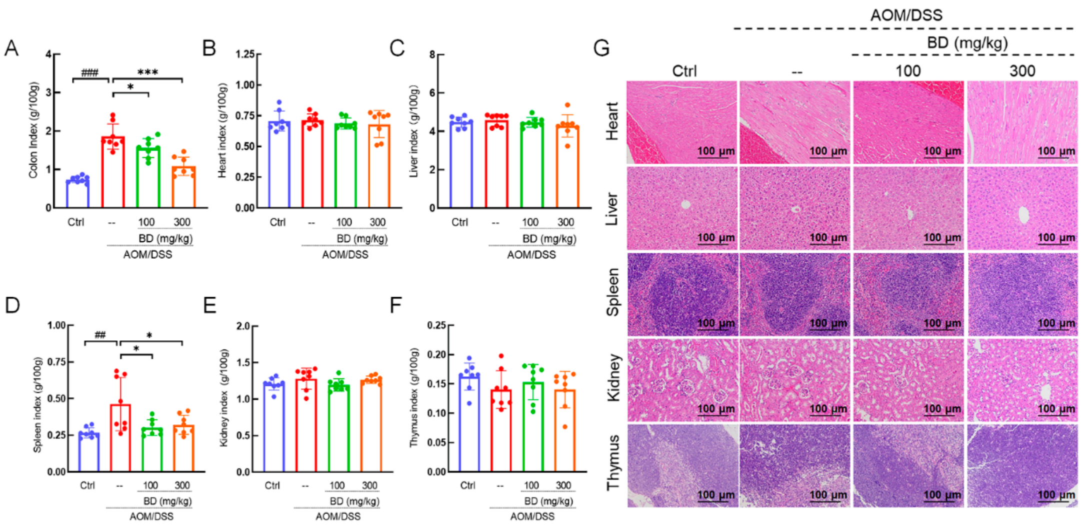

Figure S1. Organ index of (A) Colorectum, (B) Heart, (C) Liver, (D) Spleen, (E) Kidney, and (F) Thymus. (G) Histopathological analysis of the heart, liver, spleen, kidney and thymus via H&E staining (200×; scale bar: 100 μm).

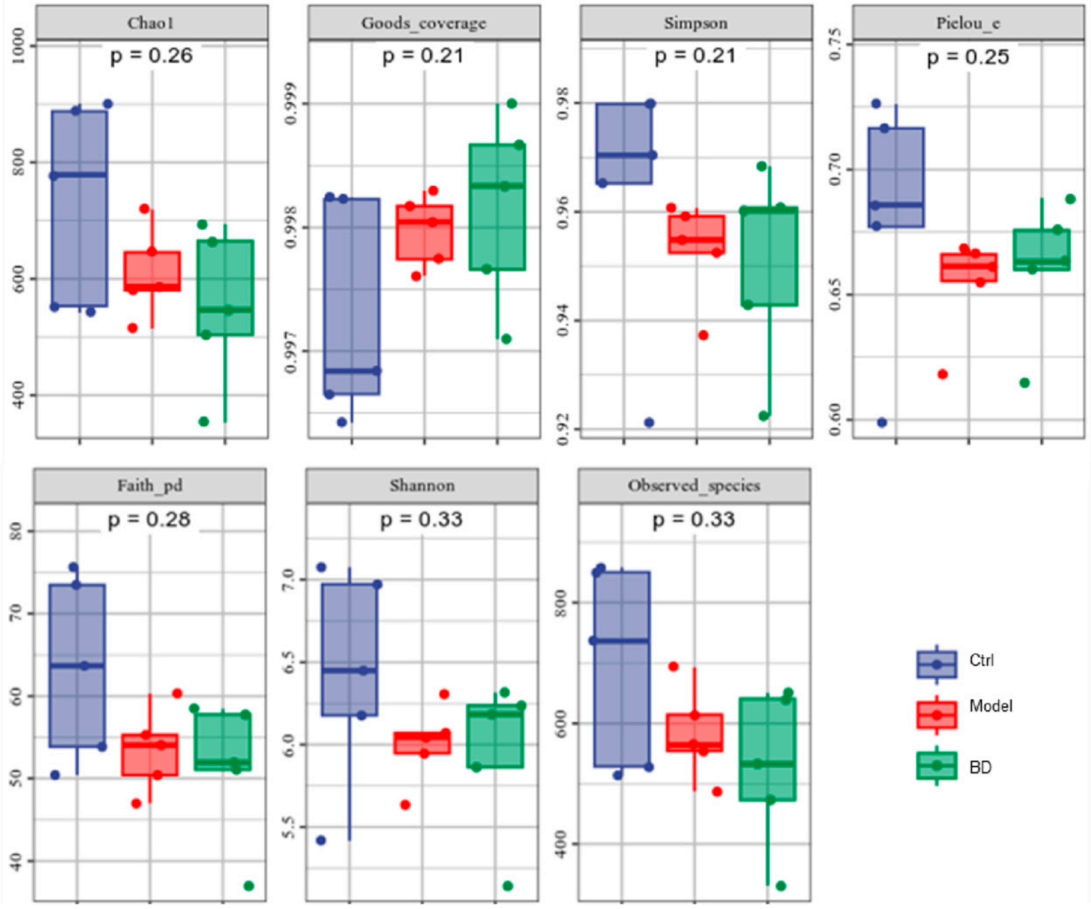

Figure S2. Alpha diversity index grouping box diagram on the intestinal microbiota in CAC mice.
